# High-Expressed Macrophage Scavenger Receptor 1 Predicts Severity Clinical Outcome in Transplant Patient in Idiopathic Pulmonary Fibrosis Disease

**DOI:** 10.1155/2021/6690100

**Published:** 2021-01-31

**Authors:** Mingfeng Zheng, Tian Tian, Jialong Liang, Shugao Ye, Jingyu Chen, Yong Ji

**Affiliations:** ^1^Department of Cardiothoracic Surgery in Affiliated Wuxi People's Hospital, Wuxi, China; ^2^Department of Dermatology, The Affiliated Sir Run Run Hospital of Nanjing Medical University, Nanjing, China; ^3^Department of Lung Transplant Center, Key Laboratory of Human Organ Transplant in Jiangsu Province, Affiliated Wuxi People's Hospital, Wuxi, China; ^4^Key Laboratory of Pathogen Biology of Jiangsu Province, Nanjing, China

## Abstract

**Background:**

Lung transplantation has been performed worldwide and admitted as an effective treatment for patients with various end-stage lung diseases. However, limit reliable clinical indicators exist to identify patients at high risk for allograft failure in lung transplant recipients. The recent advances in the knowledge of immunological aspects of the pulmonary diseases, for that innate macrophage activation, are induced by pathogen or pathogen-derived molecules and widely accepted as the critical evidence among the pathogenesis of lung inflammation and fibrosis. This study was aimed at evaluating the clinical significance of CD86- and macrophage scavenger receptor 1- (MSR1-) positive cells during the development of idiopathic pulmonary fibrosis (IPF) and pulmonary arterial hypertension (PAH), and their potential roles in the prediction of the outcomes after lung transplantation were examined.

**Methods:**

Tissues from lung transplantation for 37 IPF and 15 PAH patients from the Department of Cardiothoracic Surgery in Wuxi People's Hospital from December 2015 to December 2016 were analyzed by immunohistochemistry (IHC) for detecting the expression and CD86 and MSR1 and correlated with clinical events after lung transplantation.

**Results:**

IHC results showed that the expression of MSR1, IL-13, and arginase-1 (Arg1) but not CD86 in the lung section of IPF patients was dramatically enhanced when compared with that of PAH patients. The expression of MSR1, IL-13, and Arg1 but not CD86 in the lung from IPF patients with smoking was significantly increased when compared with that from nonsmoking subjects. In addition, the expression of MSR1-positive cells in IPF subjects with *Klebsiella pneumoniae* infection was dramatically enhanced than that in noninfection subjects. MSR1-positive macrophages were negatively associated with FEV1 and with FVC but not associated with TLC and with TL_CO_. However, CD86-positive macrophages were not significantly associated with the above lung function-related factors. Furthermore, MSR1 had a higher area under the ROC curve (AUC) than CD86 for IPF diagnosis. Survival analysis indicated that high levels of MSR1-positive macrophages had a worse prognostic effect for IPF patients with lung transplantation.

**Conclusion:**

Our study indicates the clinical significance of *Klebsiella pneumoniae* infection-related MSR1-positive cells in IPF progression, and it could be a prognostic marker in IPF after the lung transplant; development strategies to reduce the expression of MSR1-positive macrophages in IPF may be beneficial for the lung transplant.

## 1. Introduction

Idiopathic pulmonary fibrosis (IPF) is a progressive and fatal illness characterized by the development of extracellular matrix deposition resulting in severe dyspnea and impairment of lung function [1]. The median survival of patients with IPF ranges from 2.84 to 4.8 years after diagnosis, depending on the stage of the disease and histopathologic features [2–4]. The pathologic processes that cause disease progression are thought possibly related to individual genetic and nongenetic factors (e.g., pathogen infection and smoking) [5]. Pulmonary arterial hypertension (PAH) is a progressive disease of the precapillary pulmonary vasculature, which occurs when most of the very small arteries throughout the lungs narrow in diameter, resulting in resistance to blood flow and eventually right heart failure and death [6]. Despite a large spectrum of drug targets and compounds, lung transplantation is the only intervention shown to increase life expectancy for patients with IPF and severe PAH, when medical therapy is no longer effective. Thus, understanding the accurate indicators for disease progression might improve the clinical management of lung transplantation.

The prognosis of individual patient underlines that lung transplantation is difficult to predict because there are no reliable clinical parameters or biomarkers to reflect disease progression. As the most abundant immune cells in the lungs (approximately 70% of the immune cells), macrophage phagocyte pathogenic microorganisms play a central role in airway remodeling in wound healing and pulmonary fibrosis [7]. Lung macrophages display polarized phenotypes by which they can be divided into two distinct populations, alveolar macrophages (AMs) and interstitial macrophages (IMs) [8]. AMs are located on the luminal surface and initiate the immune responses in the lungs during encounter with various pathogens, while IMs are located in the lung tissue interstitium and remodel the lung tissues [9]. Evidence has pointed towards a potential role of an altered lung microbiome in triggering IPF progression [10], suggesting that some pathogens or pathogen-derived molecules have the potential roles in IPF development that is associated with lung macrophage activation [11]. Depending on the microenvironment, macrophages can be polarized into classically activated (M1) macrophages and alternatively activated (M2) macrophages [12]. M1 macrophages are activated by microbial agents and/or Th1 cytokines, such as interferon gamma (IFN-*γ*), can increase the phagocytic capacity along with the expression of costimulatory molecules (such as CD86) and secrete proinflammatory cytokines (TNF-*α*, IL-6, and IL-12), and play essential roles in clearing bacterial, viral, or fungal infections and causing tissue damage [13]. On the other hand, M2 macrophages are stimulated by Th2 cytokines and involved in tissue repair and remodeling, angiogenesis, and metabolic responses [13–15]. Macrophage polarization is implicated in promoting and regulating lung fibrosis. M1 macrophages induced by MMP28 gene knockout attenuate the bleomycin-induced lung fibrosis [16]. In addition, bleomycin-induced fibrosis is impaired in ST2-deficient mice, which is accompanied by increased M1 macrophages [17]. Pulmonary fibrosis is associated with a distinct type of M2 activation, suggesting a profibrotic role of M2 macrophages in the development of lung fibrosis [18, 19].

Previous studies showed that MSR1, one marker for M2 polarization [20], plays a critical role in the induction of inflammatory reactions and innate and adaptive immune responses by recognition of exogenous pathogen-associated molecular patterns (PAMP) and endogenous ligands [21, 22]. MSR1 plays vital roles in the process of silica-induced fibrosis, for that the MSR1-deficient mice exhibited little to deposition of collagen [23]. The expression of MSR1 was increased in unilateral ureteral obstruction- (UUO-) induced renal fibrosis [24]. It was reported that the expression of MSR1 was significantly increased in the whole blood of IPF patients [25]. Stimulation with collagen-type monomers significantly upregulated MSR1 expression in alveolar macrophages from patients with idiopathic pulmonary fibrosis, suggesting a potential role of MSR1 for the development of lung fibrosis [26]. However, little is known about the potential clinical role of such macrophage subsets in the clinical manifestations and outcomes of individual IPF patients after lung transplantation.

On the background of these findings, we became interested in investigating the activation type of macrophages from 15 IPF patients using immunochemical staining containing pulmonary arterial hypertension patients, which is evolving as an important factor that can adversely affect outcomes in chronic lung disease [27], and 37 severe IPF from December 2015 to December 2016. We assess not only the correlation between MSR1- or CD86-expressed macrophages and clinicopathological factors of lung fibrosis but also its influence on the survival after lung transplantation. We hypothesized the MSR1-positive macrophages in IPF patients could be used as a new prognostic biomarker and a potential therapeutic target for lung transplantation.

## 2. Materials and Methods

### 2.1. Study Design and Human Tissue Samples

Voluntary lung donation after the brain and cardiac death of citizens has become the sole source of organ transplants in Mainland China from January 2015 [28]. This study is approved by the ethics committee of Wuxi People's Hospital and performed in accordance with the ethical principles originating in the Declaration of Helsinki, consistent with Good Clinical Practice and applicable regulatory requirements. And these procedures and samples involved in this study were performed by surgical teams at the Department of Cardiothoracic Surgery in Wuxi People's Hospital from December 2015 to December 2016. Protocols and informed consent forms for this study were approved by appropriate institutional review boards. All patients provided written informed consent prior to participating in any study procedures.

A total of 52 patients' lung samples from 37 severe IPF patients and 15 PAH (patients having a mean pulmonary arterial pressure of greater than 25 mmHg [29]), both of which diagnosed according to the published consensus statements through combined clinical, radiological, and pathological examination [29, 30], were included in the study. Demographic and clinical data of patients with IPF and PAH patients were obtained from paper records. All the patients' key clinical characteristics and the data, such as age, sex, smoking history, and BMI, were duly matched (Table 1). No patients received disease-modifying treatment (pirfenidone or nintedanib) prior to surgery. Among these 37 IPF patients, 11 subjects were evaluated to positive infection (*Klebsiella pneumoniae*) according to previous criteria [31].

All the lung tissue samples were selected from transplant explants according to the patient's informed consent and showed features of excellent tissue preservation and adequate lung inflation. Lung resections were fixed in 4% paraformaldehyde, immersed in 20% sucrose, mounted in OCT compound, and sectioned with microtome at 5 *μ*m; then, all lung tissue samples were kept at 4°C.

### 2.2. Immunohistochemistry

After removing OCT or paraffin, all the lung tissue slides were dewaxed and hydrated using a graded series of ethanol and water. Then, antigen retrieval was performed with citrate buffer at a pH level of 6 for 5 minutes. After that, the slide was fixed with 4% paraformaldehyde and permeabilized with 0.4% Triton X-100 in PBS for 15 min at room temperature. Unspecific binding sites were blocked with PBS+2% BSA for 30 min at 4°C. Then, the rabbit anti-human CD86 antibody, rabbit anti-human MSR1 antibody (CST, 1 : 1000 dilution), rabbit anti-human IL-13 antibody (CST, 1 : 800 dilution), or rabbit anti-human arginase-1 antibody (Invitrogen, 1 : 1000 dilution) was used as the primary antibody and cocultured overnight. Then, the excess antibody was removed with PBS, and then, the slide was stained with HRP-conjugated goat anti-rabbit IgG, followed by incubation at room temperature for 30 minutes. Finally, the slices were observed under the microscope. The positive reaction of the CD86 or MSR1 protein was visible as yellow-brown staining of the cytoplasm.

Six random (100x) digital images captured from each sample were prepared for detecting the intensity of CD86- and MSR1-positive cells in the lung using Image-Pro Plus software 6.0 (Media Cybernetics, Silver Spring, MD, USA). Then, the average IOD was calculated for each sample.

### 2.3. Statistical Analysis

All statistical methods were performed using SPSS software, version 21.0 (SPSS Inc., Chicago, IL). Results are expressed as means ± standard error of the mean (SEM). Comparisons of characteristics and CD86/MSR1 expression between patients with PH and IPF subjects were performed using independent *t*-tests for normally distributed data and Mann-Whitney *U* tests for not normally distributed data. The chi-squared test was used to analyze the correlation between CD86/MSR1 expression and clinicopathologic characteristics in IPF. The levels of biomarkers were further analyzed by receiver operating characteristic (ROC) curves to determine the cut-off levels that resulted in the optimal diagnostic accuracy for each marker (CD86/MSR1) between the patients with IPF and PH subjects. Survival analysis was evaluated using the Kaplan-Meier method, and differences in outcome for each variable were evaluated using the log-rank test. *p* < 0.05 was considered statistically significant.

## 3. Results

### 3.1. Patient Demographics

Table 1 provides the details of all individuals whose lung samples were subjected to immunohistochemical staining. These parameters include number, age, sex, smoking history, pulmonary function, body mass index, and treatment. The ages of the 15 PAH subjects (59.67 ± 2.90 year old) were near-identical to those of subjects with IPF (58.35 ± 1.46, year old). The proportion of females (80.0%) among the IPF subjects was nearly the same as PAH subjects (86.5%). The proportion of subjects with smoking histories was similar among the IPF (32.4%) and PAH (26.7%) subjects (*p* = 0.68),

### 3.2. Enhanced Expression of MSR1 in IFP Patients with *Klebsiella pneumoniae* Infection

**Figure 1:**
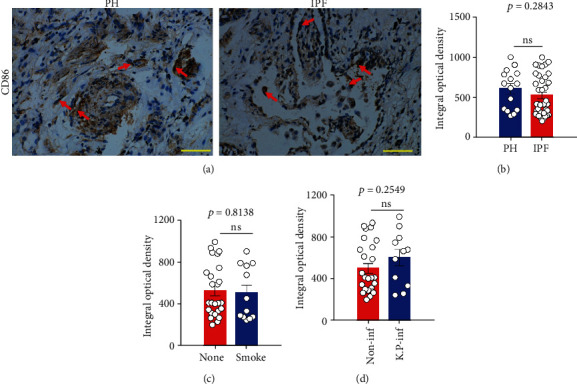
The expression of CD86 in the lung tissue samples from PH and IPF patients. (a) Representative figure of CD86 expression in the fibrotic lung in the samples from the PAH or IPF patient. Bar: 100 *μ*m. (b) The mean optical density of CD86-positive cells between PH and IPF subjects was digitized and analyzed on Image-Pro Plus software. (c) The mean optical density of CD86-positive cells in IPF subjects with (smoke) or without (none) smoke was analyzed. Data are expressed as the mean ± SEM for each group, PAH, *n* = 15; IPF, *n* = 37. ns: not significant (Student's *t*-test). (c) The mean optical density of CD86-positive cells between noninfection (non-inf, *n* = 25) and smoke subjects (*n* = 12) in IPF patients was analyzed. (d) The mean optical density of CD86-positive cells in IPF subjects with (*K.P*-inf, *n* = 11) or without *Klebsiella pneumoniae* infection (non-inf, n =26) was analyzed. ns: not significant (Student's *t*-test).

Previous studies have shown that CD86 and MSR1 are specifically expressed on macrophages and are useful as M1 and M2 macrophage markers, respectively, [32, 33]. Therefore, we performed immunostaining of CD86 or MSR1 using lung sections from IPF and PAH patients who underwent lung transplantation. As shown in Figures 1(a) and 1(b), the expression of CD86-positive macrophages in the lung sections from IPF patients was similar to that from PAH patients, suggesting that M1 macrophage polarization may not involve in the pathogenesis of IPF. In addition, there was no significant deference of CD86-positive cells in IPF subjects between with and without smoking (Figure 1(c)) or *Klebsiella pneumoniae* infection (Figure 1(d)).

**Figure 2:**
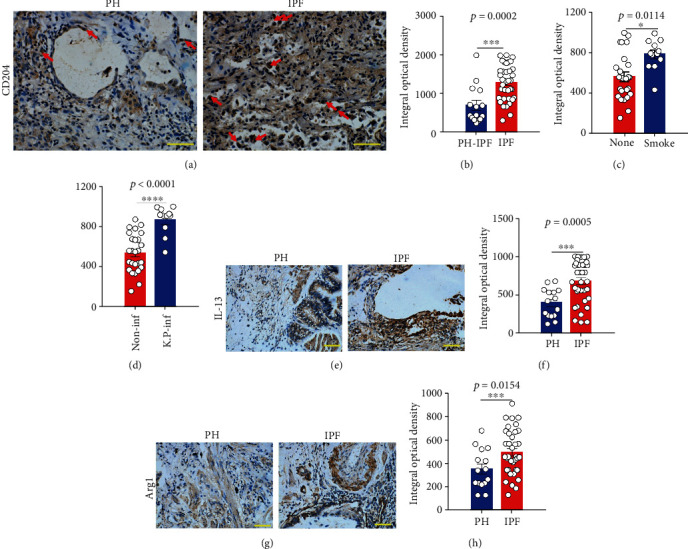
The expression of MSR1 in the lung tissue samples from PAH and IPF patients. (a) Representative figure of MSR1 expression in the fibrotic lung in the samples from a PAH or IPF patient. Bar: 100 *μ*m. (b) The mean optical density of MSR1-positive cells between PAH and IPF subjects was digitized and analyzed on Image-Pro Plus software. (c) The mean optical density of MSR1-positive cells in IPF subjects with (smoke) or without (none) smoke was analyzed. (d) The mean optical density of MSR1-positive cells in IPF subjects with (*K.P*-inf, *n* = 11) or without *Klebsiella pneumoniae* infection (non-inf, *n* = 26) was analyzed. (e, f) Representative figure of the expression of IL-13 and arginase-1 (Arg1) in the fibrotic lung in the samples from a PAH or IPF patient. Bar: 100 *μ*m. (g, h) The mean optical density of IL-13- and Arg1-positive cells between PAH and IPF subjects was digitized and analyzed on Image-Pro Plus software. Data are expressed as the mean ± SEM for each group, PAH, *n* = 15; IPF, *n* = 37. ^∗^*p* < 0.05 and ^∗∗∗^*p* < 0.001 (Student's *t*-test).

However, there was a visible significant increased expression of MSR1 expression in lung tissues from IPF patients when compared to PAH patients (Figures 2(a) and 2(b)). The expression of MSR1 in the lung tissue from smokers was significantly increased than that from nonsmokers in IPF patients (Figure 2(c)), suggesting that smoke-induced M2 macrophage polarization is associated with the development of IPF pathogenesis. Interestingly, the expression of MSR1-positive cells in IPF subjects with *Klebsiella pneumoniae* infection was enhanced than that in noninfection subjects (Figure 2(d)). Since MSR1 is the critical maker for M2 macrophages, we next detected other M2 macrophage-related factors in lung tissues from IPF patients and found that the expression of IL-13 and Arg-1 in lung tissues from IPF patients was also significantly increased in IPF patients when compared with PAH patients (Figures 2(e)–2(h)).

### 3.3. The Expression of MSR1 Is Negatively Correlated with the Lung Function in IPF Patients

**Figure 3:**
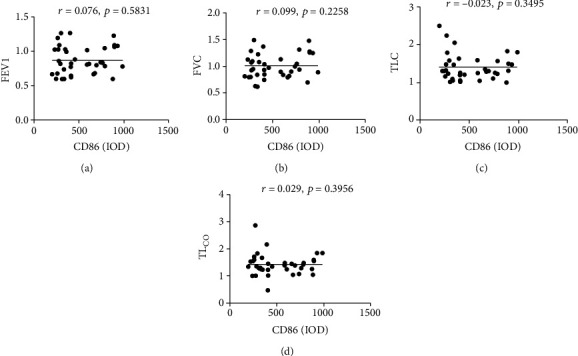
Correlation between the expression of CD86 and lung function-related factors in IPF patients (*n* = 37). (a) The correlation of CD86 and FEV1 (*r* = 0.781, *p* ≤ 0.001), (b) the correlation of CD86 and FVC (*r* = 0.734, *p* ≤ 0.001), (c) the correlation of CD86 and TLC (*r* = 0.155, *p* = 0.360), and (d) the correlation of CD86 and TL_CO_ (*r* = 0.226, *p* = 0.178) in fibrotic lungs from IPF subjects were evaluated by Pearson's chi-squared test.

**Figure 4:**
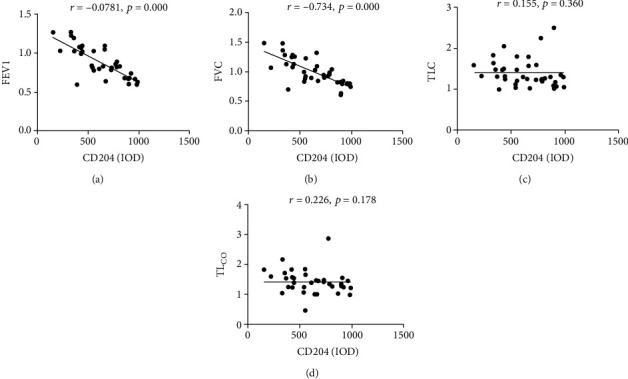
Correlation between the expression of MSR1 and lung function-related factors in IPF patients (*n* = 37). (a) The correlation of MSR1 and FEV1 (*r* = −0.076, *p* = 0.5831), (b) the correlation of MSR1 and FVC (*r* = −0.099, *p* = 0.2258), (c) the correlation of MSR1 and TLC (*r* = −0.023, *p* = 0.3495), and (d) the correlation of MSR1 and TL_CO_ (*r* = 0.029, *p* = 0.3956) in fibrotic lungs from IPF subjects were evaluated by Pearson's chi-squared test.

Next, we analyzed the connection between the expression of CD86 or MSR1 and parameters related to pulmonary function. As shown in Figures 3(a)–3(d), there was no association between the expression of CD86 and FEV1, FVC, TLC, or TL_CO_ in IPF subjects (*r* = 0.076, *p* =0.5831; *r* = 0.099, *p* = 0.2258; *r* = −0.023, *p* = 0.3495; *r* = 0.029, *p* = 0.3956). However, FEV1, and FVC were found to be significantly negative correlated to MSR1 expression in fibrotic lungs from IPF subjects (*r* = −0.781, *p* ≤ 0.001; *r* = −0.734, *p* ≤ 0.001; Figures 4(a) and 4(b)), while there were no significant correlations between the expression of MSR1 and TLC or TL_CO_ in IPF subjects (*r* = 0.155, *p* = 0.360; *r* = 0.226, *p* =0.178; Figures 4(c) and 4(d)).

### 3.4. MSR1 Expression in the Lung of IPF Patients Is Significantly Associated with the Severity of Fibrosis and Lung Function

**Figure 5:**
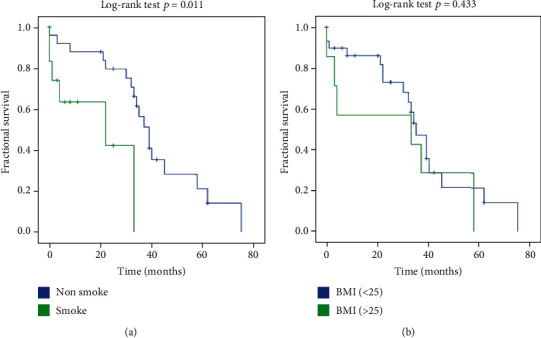
Survival curves of patients with idiopathic pulmonary fibrosis (IPF) grouped by smoking or BMI: (a) survival curves of patients with IPF nonsmoke (blue line, *n* = 25) and patients with smoke (green line, *n* = 12). (b) Survival curves of patients with IPF low BMI (blue line, *n* = 30) and patients with high BMI (green line, *n* = 7). Log-rank test was used.

The mean survival of patients with stable IPF after lung transplantation was 25.03 ± 20.02 months. We next examined the prognostic roles of smoke and BMI, which were closely related to the outcome of IPF [34, 35], in IPF patients after lung transplantation. Results showed that patients with smoke (*n* = 12) had a significantly decreased life expectancy compared with nonsmokers (*n* = 25) in IPF patients after lung transplantation (Figure 5(a); *p* = 0.011). However, no difference in survival on comparing subgroups of patients with IPF between low BMI (*n* = 30) and high BMI (*n* = 7) was observed (Figure 5(b); *p* = 0.433).

**Figure 6:**
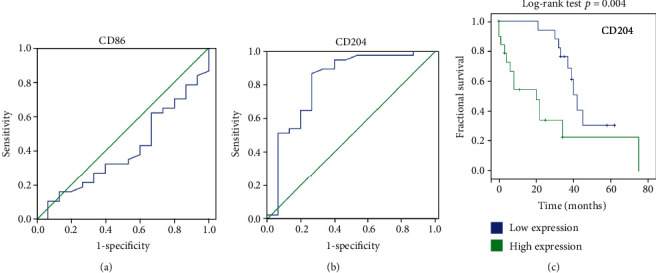
ROC and survival curves of patients with idiopathic pulmonary fibrosis (IPF) grouped by CD86 or MSR1 expression: (a, b) area under the receiver operating characteristic curve (AUC-ROC) of CD86 and MSR1 for distinguishing IPF patients from PAH; (c) survival curves of patients with IPF low expression of CD86 (blue line, *n* = 16) and patients with high expression of CD86 (green line, *n* = 21). Log-rank test was used.

To assess whether the expression of MSR1 or CD86 would predict survival after transplantation in IPF patients, ROC curve analysis was used to evaluate the discriminating capability of the two biomarkers to differentiate IPF patients from PAH subjects. Results showed that the integrated optical density (IOD) value of MSR1 ≥ 638 is the cut-off point (Figures 6(a) and 6(b)). This value represents the best compromise between the best sensitivity (90.0%) and specificity (94.1%) (MSR1: AUC = 0.921, 95% CI: 0.672-0.964, *p* < .0001). However, there is no apparent distributional difference between the CD86 values of the two groups (CD86: AUC = 0.399, 95% CI: 0.235-0.564, *p* = 0.258).

Subsequently, we divided patients into two groups according to the expression of MSR1, as demonstrated by less than 638 integrated optical density (IOD) or more than 638 IOD as low expression and high expression, respectively. The Kaplan-Meier survival analysis showed that patients with high expression of MSR1 (21 patients) had a significantly lower disease-specific survival rate than those with low expression (16 patients) (Figure 6(b); *p* = 0.004).

## 4. Discussion

In this study, we have evaluated macrophage-specific markers in the lungs from PAH patients and patients with severe-stage IPF undergoing lung transplant. Results show that M2 macrophage-related markers (MSR1, IL-13, and Arg1) are abnormally expressed in patients with IPF than PAH. Moreover, the expression of MSR1 in the lung tissues from IPF patients is also highly associated with the lung functions of afflicted individuals. Other demonstrations here are that the cases with higher numbers of M2 macrophages in the lungs from IPF patients had a poor clinical outcome after lung transplant. Our data are supported by a study on a small group of patients with IPF, who had increased MSR1 expression of alveolar macrophages from bronchoalveolar lavage [26]. There is further evidence of a potential role for M2 macrophage polarization in the fibrotic tissue response including liver fibrosis [36, 37], renal fibrosis [38], pulmonary fibrosis [16, 39], pancreatic fibrosis [40], and cardiac interstitial fibrosis [41].

Different macrophage subsets contribute important activities towards the initiation, maintenance, and resolution phase of fibrosis [42]. M2 macrophages are crucial in the pathogenesis of IPF by providing profibrogenic factors, favoring cell growth, collagen formation, and wound-healing function [43]. Gharib et al. reported that MMP28 promotes lung fibrosis associated with induction of M2 programming by using MMP28 knockout mice [16]. Another article reported that lung fibrosis induced by IL-10 overexpression increased the expression of M2 macrophages in both BAL and whole lung tissues [44]. However, the expression of CD86 (M1 macrophages) was weakly detected in/around areas of fibrosis [45]. These observations may explain the enhanced M2 but not M1 macrophage invasion into the lung in the development of fibrosis in our study. The reason why M2 macrophage-related markers are higher in IPF than in PAH patients is probably involved in many diverse mediators, including prostaglandin (PG) E2, endothelin 1 (ET-1), transforming growth factor- (TGF-) *β*, or IL-6, in the development of PH [46], which need further study in the future.

We found that the numbers of MSR1-positive macrophages were negatively correlated with the lung functions (FEV1 and FVC) in IPF patients; however, there was no association between CD86^+^ macrophages and lung functions. Kaku et al. showed similar results that M2 macrophages (CD163^+^ or MSR1^+^) had significant negative correlation with FEV1 in COPD patients [47]. Cigarette smoking has been reported to be one risk factor for idiopathic pulmonary fibrosis [48], and the previous study provided transcriptome-based evidence that macrophages likely contribute to lung disease pathogenesis due to the smoking-induced reprogramming towards M2-polarized phenotype [49]. Our data show that MSR1 expression but not CD68 in the lungs from smokers was dramatically increased compared with MSR1 expression from nonsmoking IPF patients. To the best of our knowledge, this is the first study showing a smoking-related upregulation of MSR1 expression in IPF patients. One potential mechanism involved might be endotoxin (LPS) exposure by cigarette smoking, as MSR1 is required for LPS-induced TLR4-mediated NF-*κ*B activation in macrophages [50]. In addition, smoking was reported to affect a number of biological mediators of inflammation through its effect on immune-inflammatory cells, such as neutrophils, natural killer (NK) cells, dendritic cells, and mast cells, as well as macrophages; we speculated that MSR1 is affected by the activation of the above cells [51]. Similar results of alveolar macrophages from patients with COPD were found and more CD204 was expressed in COPD smokers than non-COPD smokers and nonsmokers [52]. Consistent with this, IPF in patients with smoke history was associated with poor clinical prognoses after lung transplantation. However, the present study only illustrated the representative markers (MSR1 and CD86) in patients, while there existed various macrophage phenotypic markers, including cytokines, chemokines, soluble cytokine receptors, and other surface receptors [53], which needs further evaluation in the future. A study had indicated that the obvious pathogen infection, such as *Klebsiella pneumoniae*, *Mycobacterium tuberculosis*, and *Acinetobacter baumannii*, was associated with the IPF patients with deteriorating symptoms [31]. It is very interesting to explore the potential roles between the expression of MSR1 and the pathogen infection in IPF patients. Our data indicate that the expression of MSR1 in IPF subjects with *Klebsiella pneumoniae* infection was more than that in noninfection subjects, probably due to *Klebsiella pneumoniae*-produced various endotoxins (LPS) that may involve in MSR1 activation [50].

Although several soluble molecules and bronchoalveolar lavage fluid (BALF) marker, including CXCL11 [54], KL-6 [55], IL-7 [56], and YKL-40 [57], for IPF have been investigated, however, there is an urgent need to discover new biomarkers to predict the clinical outcome of IPF patients after lung transplantation. Only a few reports indicated that factors associated with the survival after lung transplantation for IPF have been examined, including preoperative pulmonary artery pressure [58] and lung allocation score [59]. In fact, we speculate that the microenvironment of lungs from IPF patients offers them the best chance of survival. So, looking for the potential markers in the lungs might be one meaningful prognostic indicator. When patients with IPF were determined to undergo lung transplantation, we can get adequate pathological lung tissue to examine special markers. A further question is whether posttransplant survival should be given increased or decreased weight in the pro- or anti-inflammatory macrophages, which might be affected by various pathogens and pathogen-derived molecules. Our data show that higher expression of MSR1 in the lung was associated with worse clinical prognoses in IPF patients after lung transplantation, suggesting that MSR1 might serve as one potential quantitative tool to gauge posttransplant survival.

## 5. Conclusions

In summary, our data imply that an increased MSR1, IL-13, and Arg1 expression occurs in patients with IPF, an observation that could suggest a possible role for M2 macrophages in the pathogenesis of pulmonary fibrosis. Since the expression of MSR1 correlated significantly with functional indices (FEV1, FVC) of lung fibrosis severity, it could be a useful biomarker in the assessment of the clinical status of patients with IPF. More importantly, M2 macrophages seem to be one potential disease severity marker after lung transplant, suggesting the predictive value of MSR1 in the survival.

## Figures and Tables

**Table 1:** Clinical characteristics of cases in this study.

	Pulmonary arterial hypertension secondary to IPF (PAH)	IPF patients with severe fibrosis (lung transplant patients)	*p* value
Clinical data			
*N*	15	37	
Sex	3 females; 12 males	5 females; 32 males	0.870
Age (yr) (mean ± SEM)	59.67 ± 2.90	58.35 ± 1.46	0.725
Smoking in history (percent)	26.7%(4/15)	32.4% (12/37)	0.683
BMI (mean ± SEM)	22.27 ± 0.60	22.69 ± 0.62	0.661
Lung function			
FEV1 (mean ± SEM)	1.36 ± 0.03	0.86 ± 0.04	*p* ≤ 0.001
FVC (mean ± SEM)	1.32 ± 0.02	0.99 ± 0.04	*p* ≤ 0.001
TLC (mean ± SEM)	1.69 ± 0.03	1.41 ± 0.06	*p* ≤ 0.001
TL_CO_ (mean ± SEM)	1.94 ± 0.03	1.41 ± 0.07	*p* ≤ 0.001
Medication			
Prednisolone	100%	100%	
Azathioprine	0	0	
N-Acetylcysteine	100%	100%	

Definitions of abbreviations: PAH = pulmonary arterial hypertension; IPF = idiopathic pulmonary fibrosis; TL_CO_ = diffusing capacity of carbon monoxide. ^a^ Significant differences between the groups: *p* <0.05.

## Data Availability

No data were used to support this study.

## Abbreviations

PAH: Pulmonary arterial hypertension
MSR1: Macrophage scavenger receptor 1
IPF: Idiopathic pulmonary fibrosis
IHC: Immunohistochemistry
AMs: Alveolar macrophages
IMs: Interstitial macrophages
M1: Classically activated macrophages
M2: Alternatively activated macrophages
FVC: Forced vital capacity
TLC: Total lung capacity
FEV1: Forced expiratory volume in one second
TL_CO_: Transfer factor of the lung for carbon monoxide.
